# How to empower communities to take action on improving eye health

**Published:** 2014

**Authors:** Islay Mactaggart, Paddy Ricard

**Affiliations:** Research Fellow in Disability and Global Health, London School of Hygiene and Tropical Medicine, London, UK. Islay.Mactaggart@Ishtm.ac.uk; Editor, Community Ear and Hearing Health, London, UK.

**Figure:**
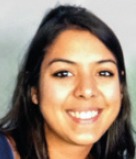
Islay Mactaggart

**Figure:**
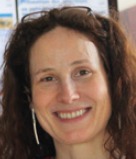
Paddy Ricard

As discussed earlier in this issue, we cannot assume, once appropriate services are established or useful information has been shared, that community eye health will automatically improve.

There are many reasons why communities may not adopt new, healthier habits or make use of recommended eye health services. These reasons are specific to each context but can be grouped into two very broad categories:

- Reasons relating to each person's beliefs and their social environment
- Practical reasons relating to access and logistics.

Working with communities can help you as an eye care provider to identify both types of barriers and to find solutions to overcome them. Indeed, while you may know **what** needs to be done from the point of view of eye heath or public health, the community will help you to work out **how** this can be done in their particular context. In this way, you can empower the community to take action to improve their eye health by listening to their views and putting them in the driving seat.

For example, early attempts to provide community-directed treatment with ivermectin in Nigeria achieved lower coverage at first, because local health workers had organised the ivermectin distribution at a time of day when the majority of working-aged adults were busy at their farms. By simply asking the community when they were more likely to be available, and changing the time of distribution accordingly, uptake improved.^1^

This article provides practical suggestions for working collaboratively with communities to improve eye health, and also briefly covers how to measure the success, or impact, of your intervention.

**Figure:**
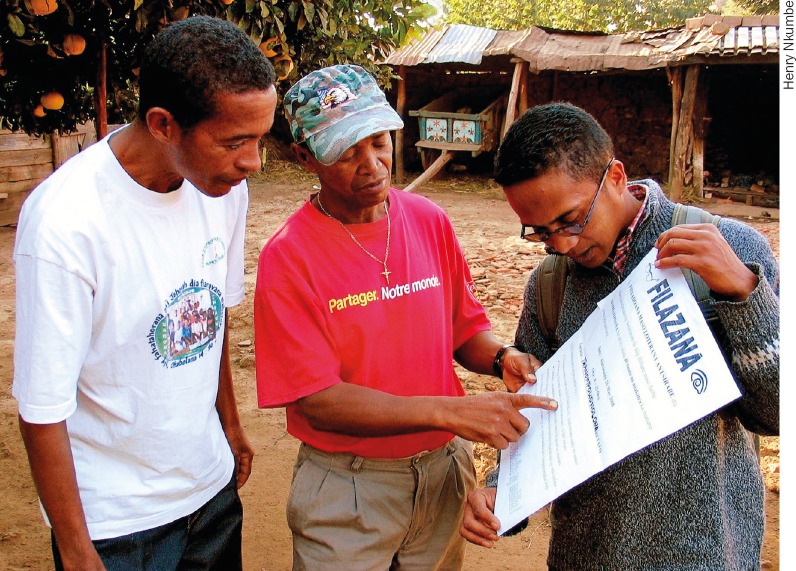
Two community eye health workers hand a local chief a poster to increase awareness about eye health. MADAGASCAR

**‘Whatever your objective is, you will stand a much better chance of achieving it if you are aware of how the community feels about eye care and eye health’**

## Identifying barriers to behaviour change with the help of the community

Any attempt to change community behaviour must begin with discussions with representatives of the community that you are targeting. This applies whether you are trying to improve uptake of services, organise mass drug distribution, or encourage a specific behaviour change such as improved sanitation to minimise risk of trachoma infection.

Whatever your objective is, you will stand a much better chance of achieving it if you are aware of how the community feels about eye care and eye health, and of any barriers or knowledge gaps that may prevent them from adopting new behaviours in this area.

### Respect the community

Establish appropriate channels of communication (e.g. meeting with village or religious leaders first, before you meet with the general community) and respect local customs regarding gatherings (e.g. a need for separate meetings for men and women in some communities).

### Include community members in your team

If the team encouraging behaviour change has no ties to or similarities with the local community/communities, this can create a divide that may prevent community members from being open about their attitudes, beliefs and practices. In the long run, this will hamper your efforts.

Conversely, involving people from the wider community as part of the team will increase the community's trust and build relationships between you and the community. Involving community members in service delivery also reduces barriers to the acceptance of new behaviours in that community. Community health workers, elder-club staff, or teachers can be involved.

If you do not speak the local language, it will also be essential from the outset to enlist the help of a reliable translator who understands what you are trying to do.

### Communicate with the community

Once you have spoken directly with leaders and gained their permission and acceptance, contact other members of the community. This can be done in many ways, such as meetings with parents, mothers, workers or through the community leaders themselves.

It is important to ask people what their needs and preoccupations are rather than simply telling them what you think they need to know. You should listen carefully and leave plenty of room for them to ask questions. Consider talking with people in groups and also at their home, as some individuals may not be comfortable expressing their concerns in public.

### Be inclusive

You will only be truly successful if all members of the community are given the relevant information and are empowered to improve their eye health. It is important to ensure that women, minority groups and people with disabilities (who may be socially excluded or face other barriers) are able to participate. People with visual impairment, in particular, should be included.

### Things to think about

- Accessible transport
- Accessible buildings
- Accessible interpersonal communication (signing, braille, audio, etc.)
- Accessible printed information (‘Easy read’, large print, use of pictures instead of words, etc.)
- Accessible non-printed information for those who are illiterate, such as using puppet shows or plays
- An inclusive attitude. Ask yourself: is anyone or any group missing here? How could we include them?

### Understand the community and identify barriers to change

It is imperative to understand **what** the community believes in relation to eye health, and **why** they believe it. Ask them what, in their opinion, would prevent them from adopting a new behaviour for improved eye health or accessing a specific service.

It is also important to seek a wider understanding of the community. While you naturally think that your health message is important, community members may have far more pressing concerns requiring their attention, or may have to deal with constraints (social, practical, etc.) that you had not anticipated.

Listening to the community, and gaining a deeper understanding of it, will improve your chances of identifying barriers that may make it difficult for the community to adopt new behaviour to improve their eye health. The panel on this page lists some questions you may want to investigate.

### Consult the community about solutions to overcome barriers

Some of the barriers may become clear as the result of discussions with community members. Others will emerge from your own understanding of the community or from a pre-tested intervention that you are planning to roll out. In both instances, the community may provide you with valuable insights on how to overcome barriers in their local context.

Put community members in charge as much as possible, as they know best what might work in their particular circumstances. Empower them to find solutions, identify individuals with leadership skills and form groups that feel positively about the proposed changes in health behaviour (or about the benefits of cataract surgery, for example); they can then influence others in the community. These groups are sometimes known as ‘coalitions of the willing’.

At the end of this process of two-way communication, you will have a much better idea of what to do to overcome barriers to behaviour change. For example, a recent project in Bangladesh provided free surgical services to children with visual and other impairments but reported that very few children came. When asked, a number of mothers expressed fear of negative surgical outcome and of travel inga lone with their child into the capital city. After group transport was organised (allowing people to travel together to the health facility) and mothers were told what would happen during and after surgery, surgery uptake increased. Mothers felt more confident and were able to support one another.^2^

**Understanding the community**

You may want to learn more about the following:

- **What** do the community believe in relation to the behaviour you are trying to change?
- **What** are the underlying reasons for these beliefs?
- **What** are community members' major concerns when it comes to eye health and general health?
- **Who** (e.g. traditional healers, religious leaders) communicates information (and sometimes misinformation) about eye health?
- **Who** in the community would make decisions relevant to the change you want to encourage (e.g. who would need to decide or be consulted regarding the building of latrines)?
- **Whose** endorsement would encourage uptake by others (e.g. village elders)?
- **What** are the daily routines of the community as a group and of individual households (e.g. when do family members work in the fields)?
- **Who** makes decisions in households (e.g. financial decisions, or decisions about what food the children eat)?

## Techniques to empower communities to take action to improve eye health

Having formed all the appropriate relationships with the community and identified (along with community representatives) what barriers exist, you need to decide how you will empower communities to improve their eye health.

The article *Techniques to encourage people to take better care of their eye health* on page 67 lists a number of specific techniques you can use to encourage behaviour change to improve eye health. It should provide a basis from which to select activities that are relevant to what you are trying to achieve. Many of the techniques listed can be used together. Indeed, it seems that the most successful programmes use 3 or 4 techniques at the same time.^3^ Each of them should be adapted to fit your context.

When deciding on the most appropriate plan of action, make sure that the techniques you select are not all of the same kind (e.g. do not rely on visual messages alone, whether via posters or videos). It is also important to choose at least one technique that allows active participation from community members.

In all cases, when creating health messages, you should:

- Use simple and practical language, be brief and avoid technical details.
- Break down information into ‘digestible’ bits that can easily be put into practice by your audience in their context. Too many recommendations given out at once will confuse, overwhelm or discourage community members.
- If needed, adapt your health message to your context. For example, state how a change of behaviour will benefit this particular community.
- Always use language that is familiar and compatible with local culture and norms, and avoid judgmental or prescriptive statements.
- Pre-test your message on a few community members or on a small community and make any adjustments needed before circulating it more widely. When collecting feedback, consider home visits alongside group discussions, as mentioned earlier.
- Although your health messages should be simple, you should provide specialists to answer more technical follow-up questions when needed (e.g. on radio phone-in shows, on a technical phone support line, etc.) This ensures that more complicated queries are adequately and appropriately addressed.

## Conclusion

Improving eye health is a complex task that depends greatly on not just your objectives, but on your own understanding of the way the community behaves. Participatory approaches – that first try to learn about the community before offering solutions – are the most successful and the most likely to improve community eye health.
